# Pathological Deficit of Cystatin B Impairs Synaptic Plasticity in EPM1 Human Cerebral Organoids

**DOI:** 10.1007/s12035-023-03812-y

**Published:** 2023-12-12

**Authors:** Amelia Pizzella, Eduardo Penna, Natalia Abate, Elisa Frenna, Laura Canafoglia, Francesca Ragona, Rosita Russo, Angela Chambery, Carla Perrone-Capano, Silvia Cappello, Marianna Crispino, Rossella Di Giaimo

**Affiliations:** 1 Department of Biology, University of Naples Federico II, Naples, Italy; 2 Department of Developmental Neurobiology, Max Planck Institute of Psychiatry, Munich, Germany; 3 Present Address: College of Osteopathic Medicine of the Pacific, Western University of Health Sciences, Pomona, CA 91766 USA; 4 Integrated Diagnostics for Epilepsy, Fondazione IRCCS Istituto Neurologico Carlo Besta, Milan, Italy; 5 Department of Pediatric Neuroscience, Fondazione IRCCS Istituto Neurologico Carlo Besta, Milan, Italy; 6 Department of Environmental, Biological and Pharmaceutical Sciences and Technologies, University of Campania “Luigi Vanvitelli, Caserta, Italy; 7 Department of Pharmacy, University of Naples Federico II, Naples, Italy; 8 Biomedical Center, Ludwig Maximilian University of Munich, Munich, Germany

**Keywords:** Cystatin B, EPM1, Human cerebral organoids, Synaptic plasticity, Extracellular vesicles, Synaptosomes

## Abstract

Cystatin B (CSTB) is a small protease inhibitor protein being involved in cell proliferation and neuronal differentiation. Loss-of-function mutations in CSTB gene cause progressive myoclonic epilepsy 1 (EPM1). We previously demonstrated that CSTB is locally synthesized in synaptic nerve terminals from rat brain and secreted into the media, indicating its role in synaptic plasticity. In this work, we have further investigated the involvement of CSTB in synaptic plasticity, using synaptosomes from human cerebral organoids (hCOs) as well as from rodents’ brain. Our data demonstrate that CSTB is released from synaptosomes in two ways: (i) as a soluble protein and (ii) in extracellular vesicles-mediated pathway. Synaptosomes isolated from hCOs are enriched in pre-synaptic proteins and contain CSTB at all developmental stages analyzed. CSTB presence in the synaptic territories was also confirmed by immunostaining on human neurons in vitro. To investigate if the depletion of CSTB affects synaptic plasticity, we characterized the synaptosomes from EPM1 hCOs. We found that the levels of presynaptic proteins and of an initiation factor linked to local protein synthesis were both reduced in EPM1 hCOs and that the extracellular vesicles trafficking pathway was impaired. Moreover, EPM1 neurons displayed anomalous morphology with longer and more branched neurites bearing higher number of intersections and nodes, suggesting connectivity alterations. In conclusion, our data strengthen the idea that CSTB plays a critical role in the synapse physiology and reveal that pathologically low levels of CSTB may affect synaptic plasticity, leading to synaptopathy and altered neuronal morphology.

## Introduction

Progressive myoclonus epilepsy type 1 (EPM1 or Unverricht-Lundborg disease OMIM 254800) is an autosomal recessive neurodegenerative disease, characterized by seizures, myoclonus, and ataxia [1]. EPM1 is mainly caused by loss-of-function mutations in the cystatin B (CSTB) gene [2, 3]. Indeed, Cstb-KO mice display some of the human EPM1 phenotypes [4, 5]. CSTB is a protein inhibiting cysteine proteases, especially cathepsin B and is ubiquitously expressed in most cell types and tissues [2, 6]. Nonetheless, the genetic removal of the target cathepsins in Cstb-KO mice does not fully rescue the pathological phenotype of these animals [7], suggesting that CSTB plays additional roles other than inhibiting cathepsins. Since the absence of CSTB leads to epileptic phenotypes, its role in the nervous system has been widely investigated. CSTB aggregates have been detected in association with senile plaque of patients suffering from Alzheimer’s and Parkinson’s diseases and in the patients with senile dementia [8, 9]. It has been observed that mouse model of amyotrophic lateral sclerosis has altered levels of CSTB [10], suggesting the involvement of this protein in neurodegenerative diseases. Moreover, CSTB protects neurons from oxidative stress [11] and prevents cerebral apoptosis [5], suggesting that it contributes to neuronal plasticity.

The adaptive ability of neurons to modulate their responses to different stimuli depends on morphological and physiological changes of synaptic contacts [12]. In this context, a highly relevant question is whether the local system of protein synthesis at synapses contributes to the plasticity by enabling the synapses to rapidly respond to stimuli independently of the cell body [13]. Interestingly, it has been demonstrated that CSTB is locally synthesized in the synaptic areas of the rat brain [14]. The link between CSTB and synaptic plasticity has been further supported by a number of experimental data showing that (i) Cstb-KO mice display altered abundance of ribonucleoproteins and ribosomal subunits in synaptic regions [15]; (ii) CSTB deficiency leads to alterations of GABAergic signaling [15, 16]; (iii) CSTB is involved in interneuron recruitment during neurogenesis, modulating the extracellular environment [17]; (iv) CSTB is secreted by nerve terminals in a depolarized-dependent way [14]; and (v) CSTB contributes to autophagy and vesicular trafficking in astrocytes [18].

Extracellular vesicles (EVs) are a heterogeneous population of vesicles produced by most cell types to be released into the extracellular space. Thereby, EVs play a key role in cell–cell communication [19]. By carrying different cargos within them, such as DNA, RNA, lipids, and proteins, EVs modify the genotypes and phenotypes of the target cells [20, 21]. In the nervous system, EV-mediated cargo transport provides a way of long-distance intracellular communication, and therefore its deregulation is often implicated in several neurodegenerative diseases [22–24]. In particular, in view of the fact that EVs are released by synaptic regions of the neurons [25, 26], this pathway may serve as a new therapeutic target for central nervous system (CNS) disorders characterized by altered synaptic plasticity.

Here, we investigated the molecular mechanisms underlying the effects of CSTB on synaptic plasticity by using cultured neurons and synaptosomal fractions derived from various experimental model systems: (i) rat brain, (ii) human cerebral organoids (hCOs) at different developmental stages, and (iii) neurons originated from neuronal progenitor cells (NPCs). We show that CSTB is released by synaptosomes not only as a soluble protein, but also in the EV-mediated pathway. Moreover, using hCOs from EPM1 patients, we demonstrate that the synaptosomal levels of presynaptic proteins and of an initiation factor linked to local protein synthesis are reduced and that the EVs trafficking is impaired. Our data suggest that alteration of synaptic plasticity due to the deficiency in CSTB is one of the key mechanisms underlying EPM1.

## Materials and Methods

### Animals

Wistar male rats (Charles River Laboratories, Calco, Lecco, Italy) of about 3 months of age were kept in the animal house at the Department of Biology, University of Naples, Italy. C57BL/6 J mice were kept in the animal facility of the Max Planck Institute of Psychiatry, Munich, Germany. The synaptosomes were prepared from C57BL/6 J mouse embryos (E18) with the day of vaginal plug, which was considered as embryonic day 0 (E0). Animals were kept with food and water ad libitum in a room with controlled temperature and a 12-h light–dark regimen (lights on at 6 AM). The adult rats were anesthetized by intra-peritoneal injection of chloral hydrate (40 mg/100 g body weight) and decapitated with a guillotine. The brains were rapidly removed, and the cerebral cortex was dissected for subsequent analyses. All the animal experimental procedures were performed in accordance with European Union guidelines.

### Generation of Human Cerebral Organoids

Reprogrammed iPSCs from human newborn foreskin fibroblasts and peripheral blood mononuclear cells isolated from two control healthy individuals and two EPM1 patients [17] were used to generate human cerebral organoids (hCOs) as previously described [27, 28]. Briefly, iPSCs were dissociated into single cells using StemPro Accutase Cell Dissociation Reagent (A1110501, Life Technologies) and plated at the concentration of 9000 single iPSCs/well into low-attachment 96-well tissue culture plates in hES medium (DMEM/F12GlutaMAX supplemented with 20% Knockout Serum Replacement, 3% ES-grade FBS, 1% nonessential amino acids, 0.1 mM 2-mercaptoethanol, 4 ng/mL bFGF, and 50 µM Rock inhibitor Y27632) in order to form embryoid bodies (EBs). On day 6, EBs were transferred into low-attachment 24-well plates in NIM medium (DMEM/F12GlutaMAX supplemented with 1:100 N2 supplement, 1% nonessential amino acids, and 5 µg/mL heparin) and cultured for additional 6 days. On day 12, EBs were embedded in Matrigel (Corning, 354,234) drops and then transferred in 10-cm tissue culture plates in NDM minus A medium (DMEM/F12GlutaMAX and Neurobasal in ratio 1:1 supplemented with 1:100 N2 supplement 1:100 B27 without vitamin A, 0.5% nonessential amino acids, insulin 2.5 µg/mL, 1:100 antibiotic–antimycotic, and 50 µM 2-mercaptoethanol) in order to form COs. On day 4, after Matrigel embedding, COs were transferred in 10-cm dishes into an orbital shaker and cultured until analysis in NDM plus A medium (DMEM/F12GlutaMAX and Neurobasal in ratio 1:1 supplemented with 1:100 N2 supplement 1:100 B27 with vitamin A, 0.5% nonessential amino acids, insulin 2.5 µg/mL, 1:100 antibiotic–antimycotic, and 50 µM 2-mercaptoethanol). COs were kept on a shaker at 37 °C, 5% CO_2_ and ambient oxygen level with NDM plus A medium, with changes of medium every 3–4 days. COs were analyzed at different months after the initial plating of the cells as indicated in the “Results” section and figures.

### Isolation of Synaptosomal Fraction from hCOs and Murine Cerebral Cortex

Synaptosomes were prepared from three different types of starting samples: (i) a pool of 20–40 organoids, (ii) cerebral cortex dissected from 3 months old rats (*n* = 3), and (iii) cerebral cortex of mouse embryos at embryonic day 18 (E18, *n* = 5). Synaptosomal preparation was made as previously described [14, 29, 30]. Briefly, cerebral cortices or hCOs were resuspended in nine volumes of cold isotonic medium (IM: 0.32 M sucrose, 10 mM Tris–HCl, pH 7.4) and homogenized with Dounce homogenizer. An aliquot of the homogenate was dissolved in RIPA buffer (50 mM Tris–HCl pH 8.8, 150 mM NaCl, 0.1% SDS, 0.5% NP-40, 0.5% DOC; protease and phosphatase inhibitor cocktail, Sigma-Aldrich). After centrifugation of the homogenate (2000 × g for 1 min, 4 °C), the sediment was resuspended in the same volume of IM and centrifuged again under the same conditions to remove the sediment containing nuclei, cell debris, and other particulates. The two supernatants were combined and centrifuged at higher speed (23,000 × g for 4 min, 4 °C) to obtain a pellet that was washed in the same volume of IM and centrifuged again as described above. The obtained pellet, named P2 fraction or crude synaptosomal fraction, contains mitochondria, myelin, and synaptosomes. Synaptosomes from adult rats and mouse embryo brain cortices were further purified by discontinuous Ficoll gradient. The P2 fraction (1 mL with protein concentration of 3.2 mg/mL) was stratified on a discontinuous gradient 5–13% Ficoll in IM (2 mL each) by centrifuging at 45,000 × g for 45 min at 4 °C. The purified synaptosomes were collected from the interface and diluted with nine volumes of IM to be re-sedimented at 23,000 × g for 20 min at 4 °C. The pellet was homogenized in IM and used for subsequent analyses. The protein content of both homogenate and synaptosomal fraction was determined by BIO-RAD method using bovine serum albumin (BSA) as the standard protein.

### Isolation of Secreted Proteins and Extracellular Vesicles from Synaptosomes

Synaptosomal fraction from hCOs was incubated in ringer medium (90 mM NaCl, 3 mM KCl, 2 mM MgCl_2_, 1 mM CaCl_2_, 1 mM glucose, 100 mM sucrose, 30 mM Tris–HCl, pH 7.5) or in depolarizing medium (43 mM NaCl, 50 mM KCl, 2 mM MgCl_2_, 1 mM CaCl_2_, 1 mM glucose, 100 mM sucrose, 30 mM Tris–HCl, pH 7.5). After 1-h incubation at 37 °C, samples were cooled on ice and centrifuged at 23,000 × g for 7 min at 4 °C. Over the resulting pellet, containing post-incubation synaptosomes, lies a supernatant containing the secreted proteins (here defined as secretome). Synaptosomes were resuspended in RIPA buffer, while the secretome was further centrifuged (100,000 × g for 2 h, 4 °C) to separate the soluble-protein fraction (SF) from the extracellular vesicles (EVs) [31]. Soluble proteins, present in the supernatant, have been concentrated by Amicon Centrifugal Filter Devices with a cut-off of 10 kDa (Merck-Millipore). Secreted EVs were resuspended in RIPA buffer for Western blot analysis (WB) or in PBS for immunostainings.

### Immunostaining of Synaptosomes and Extracellular Vesicles from Mouse Embryos

Purified synaptosomes from E18 mouse cortex were fixed in 4% paraformaldehyde (PFA) for 10 min and permeabilized with 0.3% Triton X-100 for 5 min. The samples were then incubated in the blocking solution: 0.1% Tween and 10% Normal Goat Serum (Biozol, LIN-ENG9010-10). The primary antibodies were diluted with blocking solution prior to the overnight incubation at 4 °C: synaptophysin (SYP, 1:1000, Millipore, AB9272), cystatin B (CSTB, 1:500, Antikoerper AbIN271833), and CD81 (1:500, Santa Cruz sc-166029). The synaptosomes were then washed three times in PBS by centrifugation (23,000 × g, 7 min at 4 °C), and the collected pellet was incubated with secondary antibodies (Alexa Fluor® 488 Goat Anti-Rabbit IgG (H + L) (1:1000, Life-Technologies A11008), Alexa Fluor® 546 Goat Anti-Mouse IgG (H + L) (1:1000, Life-Technologies A-11003)) for 1 h at 4 °C with gentle shaking. After centrifugation, the pellet was washed 3 times with PBS and finally resuspended in 10 µL of PBS before mounting on microscope slides to be analyzed with a Leica laser‐scanning microscope. EVs released by synaptosomes from E18 mouse cortex were stained as previously reported [32], with slight modifications. Briefly, the PBS suspension of EVs was added to an equal volume of 20% PEG1000 (Sigma, 92,897), incubated for 1 h at 4 °C with gentle shaking, and centrifuged at 3000 × g for 5 min. The pellet, containing EVs, was resuspended in PBS and permeabilized with 0.001% Triton X-100 for 5 min. The suspended EVs was added to an equal volume of 20% PEG and centrifuged at 3000 × g for 5 min and washed three times in 10% PEG. The EVs pellet was resuspended in PBS and incubated overnight with primary antibodies (CSTB 1:1000 Antikoerper AbIN271833, CD81; 1:500 sc-166029 Santa Cruz Biotechnology) at 4 °C with gentle shaking. Samples were washed three times with PEG 10% in PBS at 3000 × g for 5 min at 4 °C. Pellets were resuspended in PBS, and the secondary antibodies, Alexa Fluor® 488 Goat Anti-Rabbit IgG (H + L) (1:1000, Life-Technologies A11008) and Alexa Fluor® 546 Goat Anti-Mouse IgG (H + L) (1:1000, Life-Technologies A-11003), were added to be incubated for 1 h at 4 °C with gentle shaking. The samples were washed three times with PBS, purified from the unbound antibodies using Sephadex G-25 column (Sigma G2580), resuspended in 10 µL of PBS, and mounted on a microscope slide. Immunostained sections were analyzed with a Leica SP8 confocal laser-scanning microscope. Only signals whose dimensions do not exceed 1 µm were analyzed, according to Mondal et al. (2019) [32]. Colocalization of two proteins in the same synaptosome or EV samples was assessed by merging the different fluorescent signals on the same confocal plane. Yellow color indicates the overlap of the red and green signals.

### Gel Electrophoresis and Western Blot Analysis

Aliquots of homogenates and synaptosomal fractions were resuspended in RIPA buffer. The protein samples were denatured at 100 °C for 5 min in the sample buffer (60 mM Tris–HCl, pH 6.8, 10% glycerol, 2% SDS, 100 mM DTT, 0.1% bromophenol blue), separated in 10–15% SDS-PAGE, and transferred to PVDF membranes (Merck-Millipore). The same amount of proteins was loaded in each lane of the gel. Western blot analysis was performed as previously reported [14, 33, 34] with the following primary antibodies: SYP (1:1000, AB9272 Millipore), syntaxin (STX, 1:500, E-AB-33012 Elabscience), synaptotagmin 1/2 (SYT, 1:1000, E-AB-33005 Elabscience), CSTB (1:2000, Antikoerper AbIN271833), CD81 (1:500, AB9272 Santa Cruz), CD9 (1:500, sc-13118 Santa Cruz Biotechnology), CD82 (1:500, sc-518002 Santa Cruz Biotechnology), eIF4G2 (1:1000, HPA016965 Sigma-Aldrich), and β-actin (ACTB, 1:2000, 612,656 BD Biosciences). β-actin was used as a normalizer since its expression levels showed no substantial variations among the control and patients’ hCOs in both lysate and synaptosomal fraction (data not shown). After several washings in TBST, membranes were incubated with secondary antibody against rabbit (1:20,000, A0545, Sigma-Aldrich) or mouse (1:20,000, NA931, GE Healthcare) IgG linked to horseradish peroxidase. Signals were visualized by chemiluminescence (ECL, Millipore) on X-ray film (X-Ray Film, Fujifilm).

### Neuronal Differentiation from NPCs

Neuronal cultures were obtained from NPCs of two controls and two patient-derived cell lines [17] as previously described [35]. Neurons were cultured for 2 to 10 weeks in the neuronal differentiation medium (neurobasal medium: 1% N_2_ supplement; 2% B27 w/o vitamin A; 1% MEM-NEA; 1.25 µg/µL laminina; 1% penicillin/streptomycin, with addition of fresh 20 ng/mL BDNF, ProSpec Bio, Rehovot, Israel, 20 ng/mL GDNF, ProSpec Bio, 200 µM cAMP, Sigma-Aldrich, 1 µM ascorbic acid, Sigma-Aldrich). From week 1 to 4, the medium was changed every other day; from week 4 to 10, only half of the medium was replenished.

### Immunohistochemistry of Neuronal Cells

Young and mature neurons (2 and 10 weeks old, respectively) from NPCs differentiation were fixed with 4% PFA for 15 min at RT, washed 3 times with PBS, permeabilized with 0.3% Triton X-100 for 5 min, and incubated overnight at 4 °C in blocking solution (0.1% Tween, 10% Normal Goat Serum, Biozol, LIN-ENG9010-10). Primary and secondary antibodies were diluted in blocking solution and incubated at 4 °C overnight or 2 h, respectively. The following primary and secondary antibodies were used: SYP (1:1000, Millipore AB9272), doublecortin (DCX, 1:2000, Millipore AB2253), CSTB (1:500, Antikoerper AbIN271833), Alexa Fluor® 488 Goat Anti-Rabbit IgG (H + L) (1:1000, Life-Technologies A11008), Alexa Fluor® 546 Goat Anti-Guinea Pig IgG (H + L) (1:1000, Life-Technologies A11074), and Alexa Fluor® 647 Goat Anti-Mouse IgG (H + L), (1:1000, Life-Technologies A-21235). Nuclei were visualized using 0.5 mg/mL 4,6‐diamidino‐2‐phenylindole (DAPI) (Sigma‐Aldrich, D9542). Immunostained sections were analyzed using a Leica SP8 confocal laser-scanning microscope. The colocalization of synaptophysin and CSTB in 10-week-old neurons was confirmed by orthogonal views of the confocal pictures performed with ImageJ software. The quantification of DCX-positive nuclei on 2-week-old neuronal cultures derived from EPM1 patients and the control was performed by using “Image Calculator” and “Analyze Particles” tools of ImageJ to overlap DAPI-positive nuclei and DCX immunostainings. The parameters for threshold intensity (Otsu Dark; 21, 255) and size (30.00-Infinity) were appropriately selected to reduce the background noise and were applied to all the images analyzed. The quantification of synaptophysin-positive puncta in neuronal processes of control and EPM1 neurons was performed using the “Set Measurements” and “Total Area” tools of ImageJ to calculate the area of SYP. To display the density of SYP puncta for the number of neurons, the SYP area was normalized by the number of DCX-positive nuclei in each field analyzed.

### 3D Reconstruction of Neurons for Morphological Analysis

Mature neurons (8 weeks old), obtained from NPCs differentiation, were used for the morphological analysis. A sparse labeling of cells with GFP was obtained by lipofection of CMV-EGFP plasmid using Lipofectamine 3000 reagent following the manufacturer instructions (Thermo Fisher Scientific, L3000001). Two days after lipofection, neuronal cultures were fixed and immunostained as described above. Primary and secondary antibodies were also used: GFP (GFP-1020, 1:1000, Aves Lab) and Alexa Fluor® 488 Goat Anti-Chicken IgY (H + L) (1:1000, Life-Technologies A11039). Neurons were visualized using a Leica SP8 confocal laser-scanning microscope with × 40 objective. Z-projections with a Z-step size of 1 µm were taken to obtain 3D image. Confocal images were acquired with the Neurolucida software (MBF Bioscience, Neurolucida version 2017.03.3, 64-bit), and the subsequent tracing was performed in 3D. For the analysis of the reconstructed neurons, Neurolucida Explorer (MBF Bioscience, Neurolucida version 2017.02.9) was used. The “Branched Structure Analysis” tool was used for the neuron summary analysis and the individual process analysis. Furthermore, neuronal complexity was assessed using the Sholl analysis tool. The concentric circles were traced using a radius increment of 10 µm.

### Mass Spectrometry

Aliquots of proteins from homogenate (h) and synaptosomal fractions (syn) of 1.5-month-old control and patient hCOs were used for mass spectrometric analysis as previously described [17]. For each condition, equal amounts of proteins (80 µg in 100 µL of 100 mM triethylammonium bicarbonate) were digested with trypsin and labeled with the following TMT isobaric tags according to the procedure described elsewhere [36]: 128C and 127C for syn CTR and h CTR samples and 130N and 129N for syn EPM1 and h EPM1 samples, respectively. TMT-labeled samples were then diluted with 2% TFA to the final protein concentration of 0.5 µg/µL for the LC–MS/MS analyses. Aliquots of TMT‐labeled peptides (2.5 µg) were analyzed by high‐resolution nanoLC–tandem mass spectrometry using a Q‐Exactive Orbitrap mass spectrometer equipped with an EASY‐Spray nanoelectrospray ion source (Thermo Scientific, Rockford, IL, USA), coupled to a Thermo Scientific Dionex UltiMate 3000 RSLCnano system (Thermo Scientific, Rockford, IL, USA) as reported elsewhere [37]. The acquired raw files were analyzed with the Thermo Scientific Proteome Discoverer 2.1 software (Thermo Scientific, Rockford, IL, USA) using the SEQUEST HT search engine. The HCD MS/MS spectra were searched against the Homo sapiens Uniprot_sprot database (release 2019_11, 20,380 entries) by using the following parameters: trypsin (full) as digestion enzyme with two missed cleavage sites; mass tolerances, 10 ppm and 0.02 Da, for precursor and fragment ions, respectively; dynamic modification, methionine oxidation (+ 15.995 Da); static modifications, carbamidomethylation of cysteine (+ 57.021 Da); and the TMT label on lysines and the N‐terminus (229.1629). False discovery rates (FDRs) for peptide spectral matches (PSMs) were calculated and filtered using the Percolator node in Proteome Discoverer that was run with the following settings: maximum delta Cn 0.05, a strict target FDR of 0.01, a relaxed target FDR of 0.05, and validation based on *q*‐value. Protein identifications were accepted when the protein FDR was below 1% and when present in at least two out of three replicate injections with at least two peptides. Proteins with fold change values syn/*h* ≥ 1.2 and ≤ 0.8 were considered for bioinformatic analyses analyzed. Proteins identified by mass spectrometry were assigned to Gene Ontology (GO) biological process groups using STRING (string-db.org), version 11.5 with default parameters and in reference to the Homo sapiens as organism.

### Statistical Analyses

All the statistical analyses in this study were performed using GraphPad Prism 8 software. Data were expressed as “mean ± SEM or ± SD,” as indicated in the legends. Differences between groups were compared by the *t*-test, Mann–Whitney test, or Sidak test as indicated in the figure legends. Differences were considered statistically significant if *p* < 0.05.

## Results

### Subcellular Distribution of Synaptic Proteins in hCOs at Different Maturation Stages

**Fig. 1:**
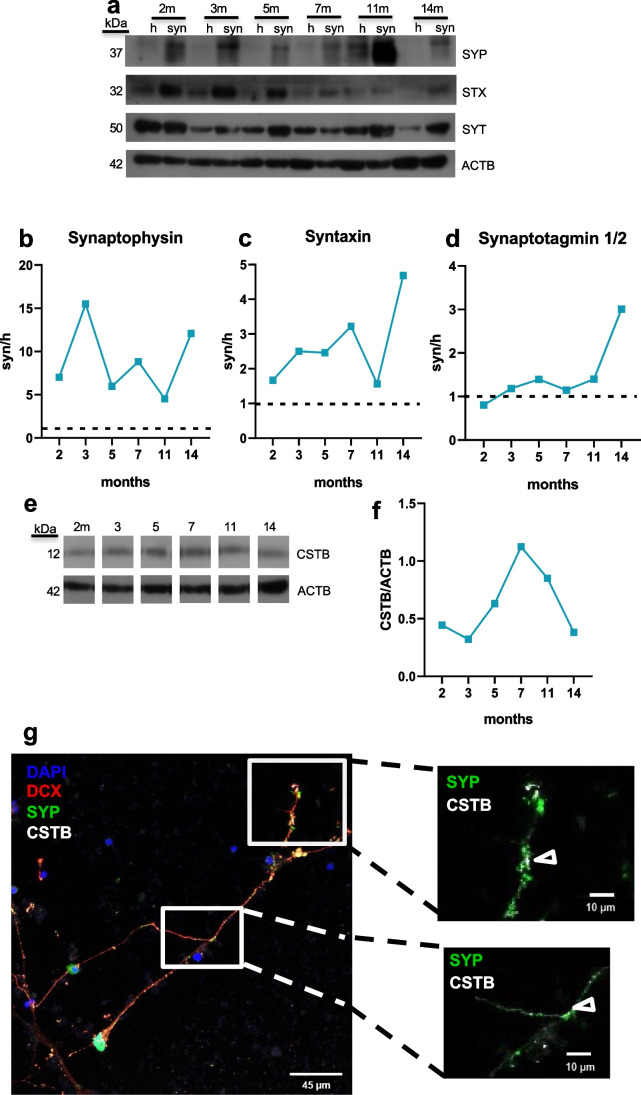
Synaptic proteins in human cerebral organoids and neurons. **a** Western blots of the total lysate (h) and synaptosomes (syn) from hCOs using synaptophysin (SYP), sintaxin (STX), synaptotagmin 1/2 (SYT), and β-actin (ACTB) antibodies. Molecular weight in kDalton (kDa) is indicated on the left. Enrichment of **b** synaptophysin, **c** syntaxin, and **d** synaptotagmin 1/2 protein level, normalized on β-actin, in the synaptosomes (syn) compared to the homogenate (h, dotted line) of hCOs at 2, 3, 5, 7, 11, and 14 months (m). **e** Western blot of the synaptosomes from hCOs using cystatin B (CSTB) and ACTB antibodies. Molecular weight in kDa is indicated on the left. **f** CSTB protein level, normalized on β-actin, in synaptosomes of hCOs. m, months. Each time point is a pool of 20–40 hCOs. **g** Micrographs showing doublecortin (DCX, red), cystatin B (CSTB, white), and synaptophysin (SYP, green) in 10-week neurons from NPCs. DAPI (blu) stained nuclei. The arrowheads in the enlarged images of the boxed area indicate the colocalization of SYP and CSTB. Scale bar in each panel

We isolated synaptosomal fractions from homogenate of hCOs at different maturation stages, as previously described [14]. To better characterize these synaptic fractions, we analyzed the levels of well-known synaptic hallmark proteins such as synaptophysin, syntaxin, and synaptotagmin 1/2 in the synaptosomes and compared them with the corresponding homogenates. In all the maturation stages analyzed, the expression level of synaptophysin, a prominent synaptic vesicles protein, was higher in synaptosomes than in the homogenate (Fig. 1a, b). Instead, the synaptosomal enrichment of syntaxin, a SNARE protein of the presynaptic membrane, was more pronounced at the late hCOs stages (14 months) (Fig. 1a, c). On the other hand, synaptotagmin 1/2, the calcium-sensor of the synaptic vesicles, was highly enriched in the synaptosomes only at 14 months, while at earlier maturation stage, no difference was observed between synaptosomes and homogenate (Fig. 1a, d). Altogether, these data indicate that synaptic vesicles are present in the synaptosomal fractions starting from early stage of maturation and suggest that synapses become physiologically active, i.e., enriched with proteins associated with exocytosis, only at late maturation stages.

We also investigated the CSTB expression levels in the synaptosomes of hCOs at different maturation stages. The result showed that the synaptic CSTB levels increased starting at 5 months, reached its peak at 7 months, and eventually decreased by 11–14 months (Fig. 1e, f). These data suggest that levels of CSTB in synapses are finely regulated during hCOs maturation. The presence of CSTB in human synaptic terminals was confirmed in 10-week neurons obtained from NPCs [35], where we observed colocalization of synaptophysin and CSTB (Fig. 1g).

### CSTB is Secreted from Rodents Synaptosomes via Extracellular Vesicles and as a Soluble Protein

**Fig. 2:**
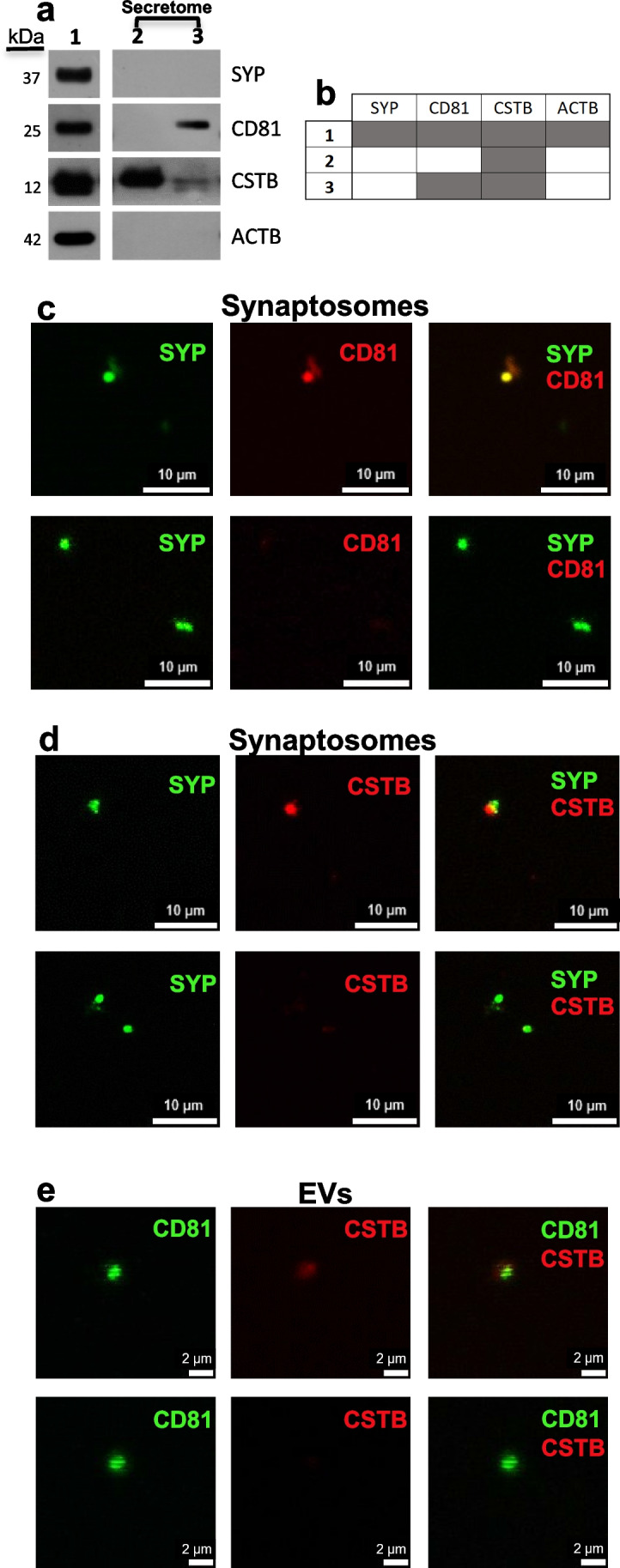
CSTB is secreted through extracellular vesicles from rodent synaptosomes. **a** Western blots of rat brain synaptosomes and their secreted fraction (secretome), using synaptophysin (SYP), cystatin B (CSTB), CD81, and β-actin (ACTB) antibodies. Lane 1: synaptosomes; lane 2: secreted soluble fraction; lane 3: secreted extracellular vesicles (EVs) fraction. Molecular weight is indicated on the left (kDa). The image is representative of experiments performed on samples from *n* = 3 rats. **b** Table showing the presence/absence of each protein in each fraction, as obtained from *n* = 3 rats. Row 1: synaptosomes; row 2: secreted soluble fraction; row 3: secreted extracellular vesicles fraction (EVs). Dark gray box indicates presence, white box indicates absence. **c** Micrographs of synaptosomes isolated from E18 mouse brain (*n* = 5) identified by synaptophysin (SYP, green) immunostaining; CD81-positive (red, upper panels) and -negative (lower panels) synaptosomes. **d** Micrographs of synaptosomes isolated from E18 mouse brain identified by synaptophysin (SYP, green) immunostaining; CSTB-positive (red, upper panels) and -negative (lower panels) synaptosomes. **e** Micrographs showing CD81-positive (green) EVs secreted from E18 mouse brain synaptosomes; CSTB-positive (red, upper panels) and -negative (lower panels) EVs. Scale bar in each panel

To better investigate the role of CSTB in the synaptic area and its possible involvement in cell–cell communication [17], we studied the secretion modality of CSTB from synapses. We previously demonstrated that CSTB is secreted from the rat synaptosomal fraction [14]. Mouse brain synaptosomes release EVs [26], and EVs secretion is known to play a crucial role in synaptic plasticity [38]. Therefore, we investigated if CSTB is secreted from rat brain cortex synaptosomes through EVs. Our results showed that the synaptosomes contain the presynaptic marker synaptophysin, the tetraspanin protein CD81 used as EVs marker, and CSTB (Fig. 2a, b). As expected, the purified EVs fraction secreted by synaptosomes is negative for synaptophysin and positive for CD81 and CSTB (Fig. 2a, b). Interestingly, CSTB is present also in the soluble secreted fraction (Fig. 2a, b), which is deprived of CD81 as expected. These results indicate that CSTB is secreted from rodents synaptosomes not only as a part of EVs, but also as a soluble protein.

To broaden our investigation to the embryonic stages, we isolated synaptosomes from E18 mouse embryonic cortices. We found that 37% of SYP-positive synaptosomes were CD81-positive (Fig. 2c), and 43% were CSTB-positive (Fig. 2d). Interestingly, we found that some of CD81-positive EVs secreted by these synaptosomes were also CSTB-positive (Fig. 2e), confirming the synaptic secretion of CSTB via EVs during embryonic development.

### Distribution and Release of EVs Proteins and CSTB from hCOs Synaptosomes

**Fig. 3:**
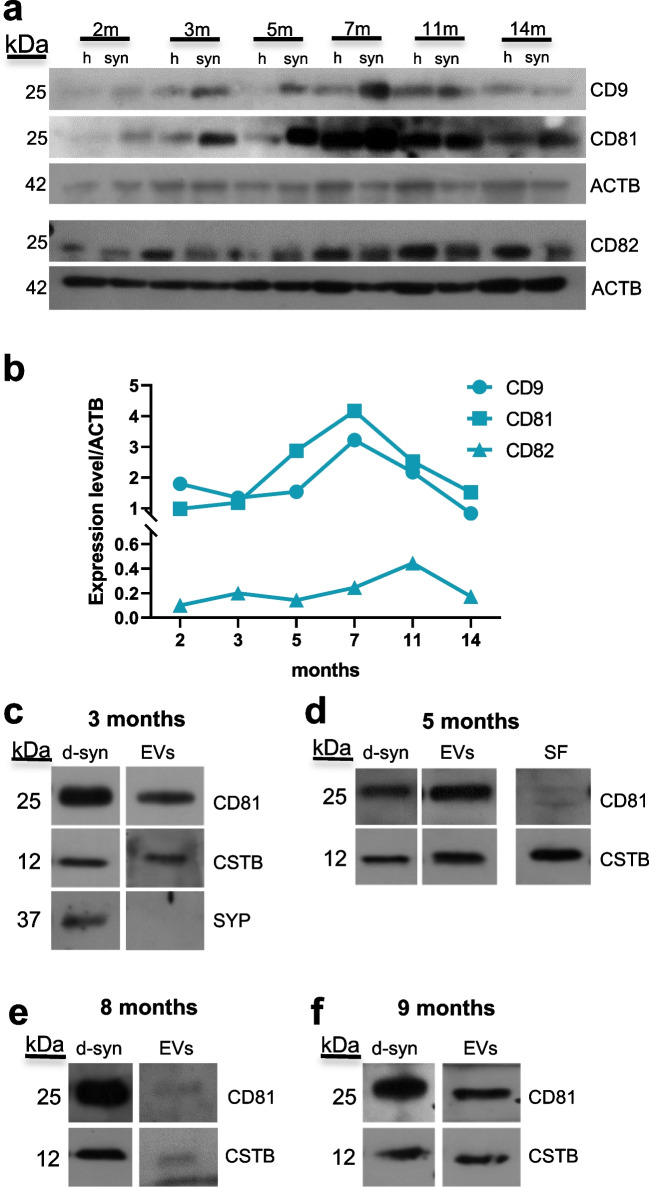
Distribution and release of EVs proteins and CSTB from hCOs synaptosomes. **a** Western blots of the total lysate (h) and synaptosomes (syn) from 2, 3, 5, 7, 11, and 14-month-old hCOs using antibodies for the EVs markers CD9, CD81, and CD82 and β-actin (ACTB). **b** Quantification of the expression levels of CD9, CD81, and CD82, normalized on the corresponding β-actin. **c**–**f** Western blots of proteins from the synaptosomal fractions upon incubation in depolarizing media (d-syn) and their secreted extracellular vesicles (EVs) and soluble fraction (SF), using CD81, cystatin B (CSTB), and synaptophysin (SYP) antibodies. Synaptosomal samples from hCOs at different maturation stages: **c** 3 months, **d** 5 months, **e** 8 months, and **f** 9 months. Each time point is a pool of 20–40 hCOs. Molecular weight of each protein is indicated on the left (kDa). m, months

To test if the synaptic fraction obtained from hCOs can be a good model for studying synaptic EVs release, we first assessed the presence of EVs markers such as CD9, CD81, and CD82 during hCOs maturation. These three markers were present at all the maturation stages analyzed (Fig. 3a, b). Specifically, CD9 showed its peak at 7 months, followed by a rapid decrease at 14 months. The trend was similar for CD81, with the peak at 7 months. Instead, CD82 displayed a different profile of expression, with a much delayed peak at 11 months (Fig. 3a, b). In conclusion, EVs markers are expressed in the hCOs synaptosomal fractions from early to late stages of maturation. Interestingly, the expression of CSTB during hCOs maturation (Fig. 1e, f) resembles CD81 and CD9 profiles more than CD82.

To investigate the EVs secretion from hCOs synaptosomal fraction, we incubated these synaptosomes for 1 h in a depolarizing medium and collected secreted soluble proteins and EVs. In the samples obtained from hCOs at different maturation stages (3, 5, 8, and 9 months), we observed that EVs fraction is CD81-positive at all stages (Fig. 3c–f). CSTB was present in secreted EVs at all maturation stages, suggesting an important role of this protein in cell–cell communication during development. Interestingly, CSTB, unlike CD81, was also detected in the soluble fraction secreted by 5-month hCOs synaptosomes (Fig. 3d), confirming that the release of CSTB in the extracellular medium is both vesicle- and non-vesicle-mediated.

### Pathological Low Expression Level of CSTB is Linked to Synaptic Impairment in EPM1 hCOs

**Fig. 4:**
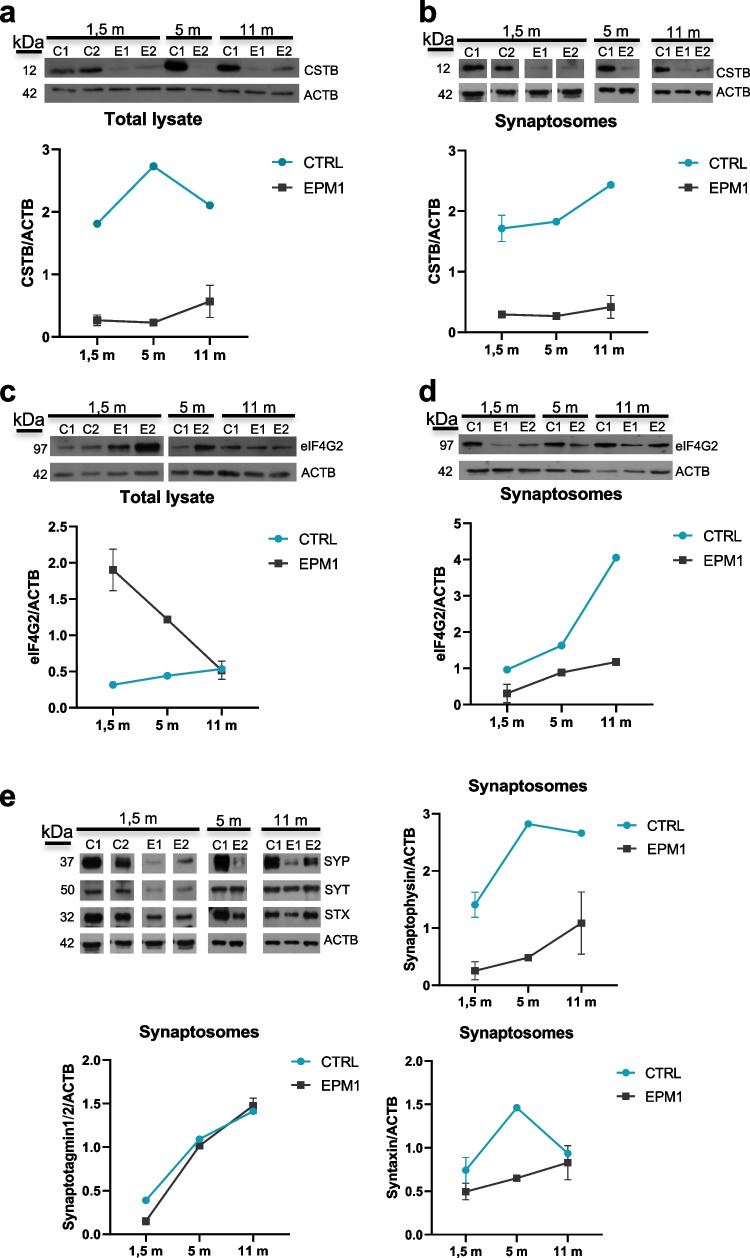
Pathological low expression levels of CSTB results in synaptic impairment in EPM1 hCOs. Western blots of **a** total lysate and **b** synaptosomal fractions from controls and EPM1 hCOs at different developmental stages using cystatin B (CSTB) and β-actin (ACTB) antibodies. Western blots of **c** total lysate and **d** synaptosomal fractions from controls and EPM1 hCOs using eukariotic Initiation Factor 4G2 (eIF4G2) and ACTB antibodies. Western blots (upper panels) and relative quantifications (lower panels). **e** Western blots of synaptosomes from controls and EPM1 hCOs at different developmental stages, using synaptophysin (SYP), sintaxin (STX), synaptotagmin 1/2 (SYT), and ACTB antibodies and relative quantifications. Molecular weight of each protein is indicated on the left (kDa). Data are presented as mean ± SD from *n* = 2 pools of 20–40 organoids. Controls: C1, C2; patients: E1, E2; m, months

Taking advantage of the cerebral organoids as a model for studying neurological diseases in humans, we isolated synaptosomes from hCOs samples obtained from EPM1 patients and control [17]. We observed that CSTB level is significantly reduced not only in the protein extracts of EPM1 hCOs (Fig. 4a), but also in the corresponding synaptosomes (Fig. 4b). A key role of CSTB in synaptic plasticity is indicated by its local synthesis and secretion from synaptic terminals [14] and by its involvement in vesicles trafficking [16]. Therefore, we hypothesized that the low level of CSTB observed in synaptic areas of EPM1 hCOs may affect synaptic functions and EVs release. To test this idea, we investigated the synaptosomal expression level of the eukaryotic initiation factor eIF4G2, which is a component of the protein synthesis machinery and is itself locally synthesized in the synaptic area [39]. We observed a decrease of eIF4G2 in the EPM1 synaptosomes compared to the levels in the control (Fig. 4d). It is noteworthy that this decrease is specifically ascribed to the synaptic area and is not generalized to the corresponding total protein extract (Fig. 4c). These results suggest that depletion of CSTB leads to an impairment of the synaptic system of protein synthesis that has been demonstrated to be crucial for the plasticity of the nerve terminals [13]. To further investigate the alteration of synaptic area in the hCOs of EPM1 patients, we also evaluated the synaptosomal expression levels of synaptic hallmarks. Interestingly, synaptophysin was observed to be reduced in EPM1 synaptosomes at all maturation stages analyzed (Fig. 4e), suggesting a deficit in the amount of synaptic vesicles. The analysis on the other two presynaptic markers, syntaxin and synaptotagmin 1/2, revealed a reduction confined to the early onset of maturation, while their expression level is comparable to the hCOs of the control at later maturation stages (Fig. 4e).

**Fig. 5:**
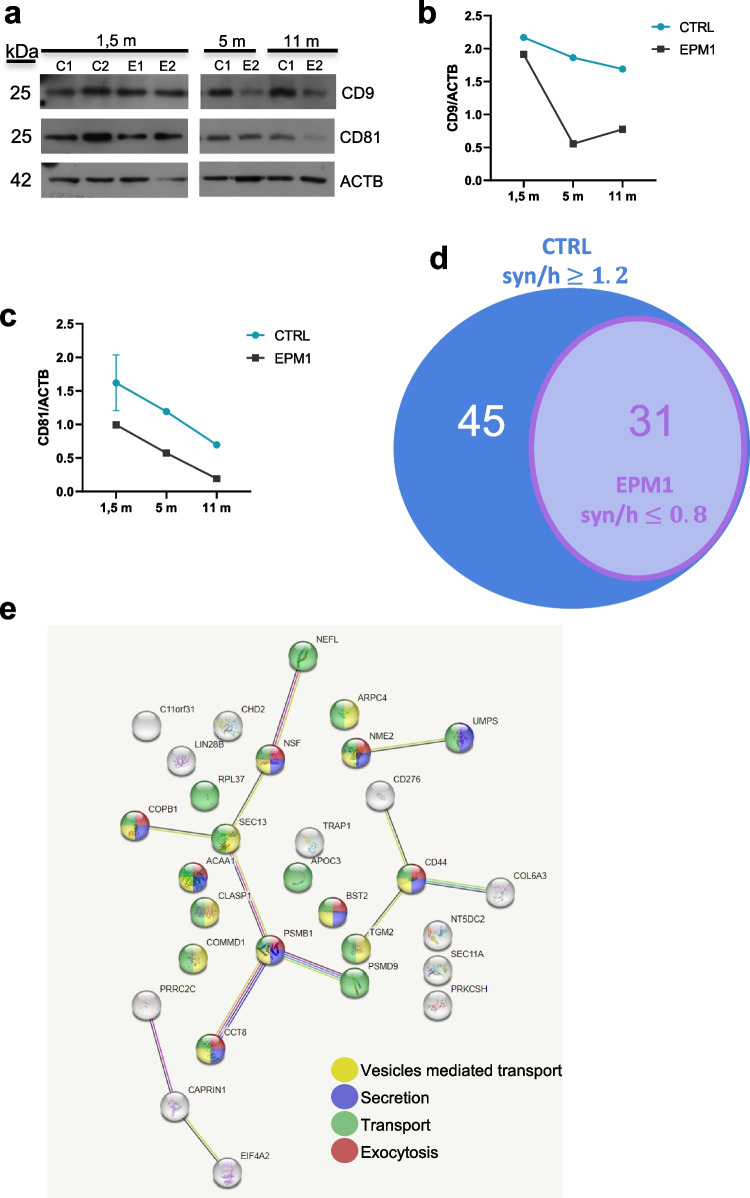
Altered expression levels of EVs markers in synaptosomes from EPM1 hCOs. **a** Western blots of synaptosomal proteins from controls and EPM1 hCOs at different developmental stages using CD9, CD81, and β-actin (ACTB) antibodies. Quantifications of the expression levels of **b** CD9 and **c** CD81 normalized on ACTB. Data are presented as mean ± SD from *n* = 2 pools of 20–40 organoids. Controls: C1, C2; patients: E1, E2; m, months. **d** Venn diagram of the proteins identified by mass spectrometry enriched in the synaptosomal fraction (syn) from 1.5-m control hCOs (CTRL) (76 proteins, syn/h ≥ 1.2) and depleted in EPM1 synaptosomes (31 proteins, syn/h ≤ 0.8). **e** Gene Ontology analysis of the 31 proteins depleted in EPM1 synaptosomes, using String database. h, homogenate

Interestingly, we also observed in synaptosomes from EPM1 patients a significant reduction of the expression levels of EVs markers such as CD9 and CD81, which further supports the idea that the deficiency in CSTB leads to dysfunctions of synaptic terminals and that the impairment of the synaptic secretion machinery comprises an important part of the pathology (Fig. 5a–c).

This hypothesis was further supported by the results of mass spectrometric analysis of: (i) protein extracts from control and patients hCOs (hom) and (ii) proteins from the corresponding synaptosomal fractions (syn). In control hCOs, we identified a set of 76 proteins enriched in the synaptosomal fraction (ratio syn/hom ≥ 1.2) (Fig. 5d and Supplementary Table 1), and thus we investigated their abundance in the synaptosomes of the hCOs derived from EPM1 patients. Intriguingly, none of these 76 proteins was enriched in the synaptosomes derived from EPM1 patients. Instead, 31 of these 76 proteins are expressed at lower level in patients synaptosomes compared to the total protein extract (ratio syn/hom ≤ 0.8) (Fig. 5d and Supplementary Table 1). Gene ontology analysis performed using the String database (string-db.org) on these 31 proteins indicated that they are involved in pathways related to secretion and vesicles-mediated transport (Fig. 5e).

In EPM1 hCOs, only nine proteins were enriched in the synaptosomal fraction (ratio syn/hom ≥ 1.2), and all of them were expressed at lower level in the synaptosomal fraction from controls (ratio syn/hom ≤ 0.8), supporting the idea that the protein composition of the synaptic area of hCOs derived from EPM1 patients is profoundly altered (Supplementary Table 1). It is noteworthy that among them, we detected two proteins, ERGIC and GBF1, which are involved in vesicular trafficking and secretion. Altogether, these results strongly suggest that CSTB is involved in EVs trafficking and release at the presynaptic bouton and that deficiency of CSTB contributes to deregulation of the synaptic functions.

### Alteration of Neuronal Morphology in the EPM1 Neurons

**Fig. 6:**
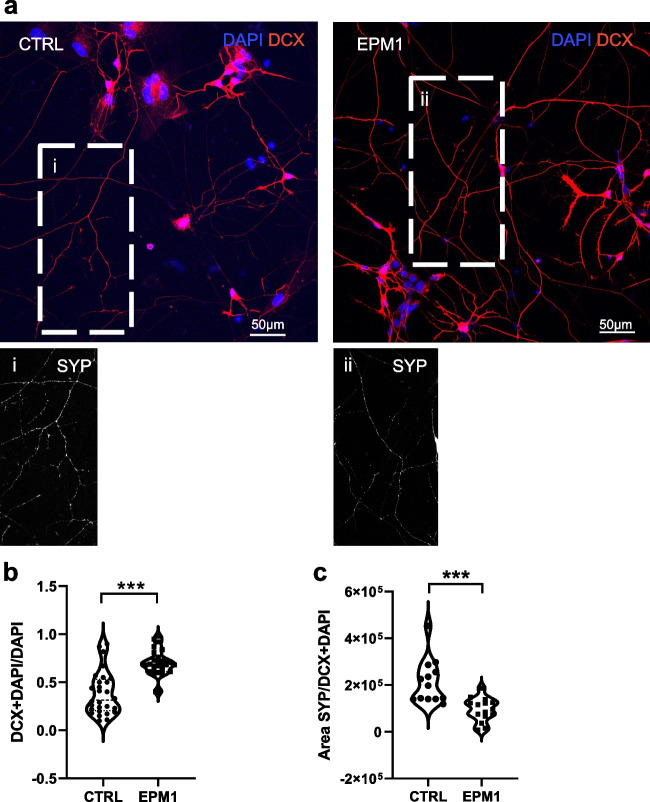
Expression level of DCX and SYP in control and EPM1 neurons. **a** Micrographs showing doublecortin (DCX, red) immunolabeling in 2-week-old neurons. Nuclei stained with DAPI (blu). Inserts showing synaptophysin (SYP, white) immunolabeling in CTRL (i) and EPM1 (ii) neurons. Quantification of **b** DCX positive nuclei and of **c** SYP positive areas. Every dot refers to a field of view from three independent CTRL and EPM1 neuronal samples. Statistical significance is based on Mann Whitney tests. ***p < 0.0001. Scale bar in each panel

**Fig. 7:**
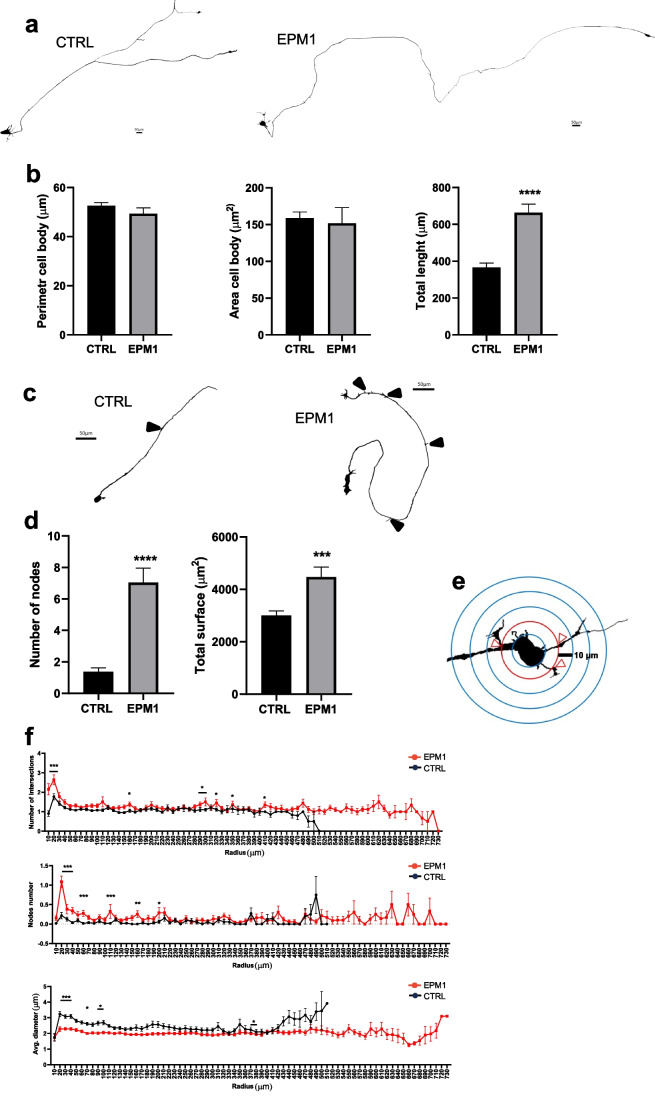
Morphological analysis of control and EPM1 neurons. **a** Morphology of representative control- (CTRL) and patient-derived (EPM1) 8-week-old neurons reconstructed with Neurolucida software. Scale bar 50 µm. **b** Quantification of the cell body perimeter, area, and total length of neuronal processes. **c** Morphology of representative control- (CTRL) and patient-derived (EPM1) 8-week-old neurons reconstructed with Neurolucida software. Arrows indicate nodes. Scale bar 50 µm. **d** Quantification of the number of nodes and total surface of neurites of control and EPM1 neurons. **e** Scheme of the Sholl analysis to measure neuronal complexity. Concentric circles with a radius increment of 10 µm were selected. **f** Number of intersections, nodes, and average diameter related to the distance from the soma of control- and patient-derived neurons. Data are presented as mean ± SEM for *n* = 53 for CTRL and *n* = 48 for EPM1 neurons. **b**, **d** Statistical significance is based on nonparametric test Mann Whitney (***p value < 0.0004, ****p value < 0.0001) or two-way ANOVA followed by Sidak’s multiple comparisons test (*p value < 0.05, **p value < 0.01, ***p value < 0.0004)

The synaptic alteration observed in EPM1 hCOs was confirmed in neuronal 2D model. By immunostaining, we analyzed neurons differentiated from hNPCs and cultured for 2 weeks. We observed an increased number of DCX-positive cells (Fig. 6a, b) among EPM1 neurons, in line with previous data [17], and a decrease of synaptophysin-positive puncta in neuronal processes of EPM1 neurons (Fig. 6a, insets a and b and Fig. 6c). These results prompted us to investigate the potential alteration of neuronal morphology in mature EPM1 neurons. We used Neurolucida imaging system to analyze GFP-sparsely-labeled 8-week-old neurons and to perform their 3D morphological reconstruction (Fig. 7). Interestingly, while there was no appreciable morphological changes in the cell body of EPM1 neurons, the neurites of these neurons were strikingly different from those of the controls in shape and number (Fig. 7a, b). Specifically, EPM1 neurons exhibited significantly longer (total length, Fig. 7b) and more branched neurites (nodes, Fig. 7c, d) compared to controls, suggesting a more complex neuronal morphology and consequently altered connectivity, which may be associated with the pathology. Indeed, increased neuronal complexity in EPM1 neurons was confirmed by Sholl analysis indicating a higher number of intersections and nodes in EPM1 neurons, especially in the first 200 µm away from the soma (Fig. 7e, f). Interestingly, although EPM1 neurites are longer and more branched than controls, they were significantly thinner (Fig. 7f).

## Discussion

The aim of our work was to investigate the role of CSTB in synaptic physiology. The involvement of CSTB in synaptic plasticity was demonstrated by our previous results showing that the protein is present in synaptosomes isolated from rat and mouse brain cortex, and it is also secreted and locally synthesized. Interestingly, for the first time, it was also observed that CSTB is present in synaptosomes from hCOs, extending our results to a human model [14].

In the present study, we investigated the protein composition of synaptosomal fraction from hCOs at different stages of development. In the early stages (1.5 month), progenitor cells differentiate to young neurons, and in the later stages (14 months), neurons are mature and physiologically active [40, 41]. In our study covering this whole period, we demonstrated that synaptosomes of hCOs are enriched in synaptic proteins and that CSTB is present at all developmental stages and peaks at the 7th month. The presence of CSTB in the synaptic areas was confirmed by colocalization with the presynaptic marker synaptophysin in the neurons generated in vitro from the progenitor cells. The different age-related expression profiles of synaptic proteins indicated that only at the later maturation stages do hCOs synapses become physiologically active, i.e., particularly enriched with syntaxin and synaptotagmin 1/2, proteins that are directly involved in vesicles docking and calcium-dependent exocytosis, respectively [42, 43]. Instead, the prominent synaptic protein synaptophysin, which is not essential for the basic processes of exocytosis and endocytosis [42, 43], was continuously enriched in the synaptosomes throughout all the developmental stages.

Moreover, we found that the synaptosomes from hCOs contain EVs markers such as CD9, CD81, and CD82 at all stages of hCOs maturation in culture, although with different age-related expression profiles. Intriguingly, the peak of expression of CSTB in synaptosomes of hCOs at 7 months of development matches the peak of the accumulation of CD81 and CD9. A step forward to deciphering the role of CSTB was the demonstration that at least some of the EVs secreted from embryonic mouse brain synaptosomes contain CSTB, as shown by its colocalization with CD81 in the EVs fraction.

It is well known that EVs secreted by neurons are able to modify the neurogenic niche to make the microenvironment more appropriate for neurogenesis and circuit assembly. Accordingly, the altered EVs secretion has a functional relevance to the neurodevelopmental diseases [44, 45]. It should also be noted that secretion of CSTB has been proposed to be important in remodeling the extracellular space for neurons by promoting tangential migration and recruitment of interneurons [17]. Here, we demonstrated a dual secretion mechanism of CSTB from synaptosomes: in a vesicles-dependent and -independent way, opening new perspectives on the biological role of CSTB as a long- and short-distance signaling cue that influences neurogenesis by affecting the microenvironment of the synapse.

The availability of hCOs generated from two different EPM1 patients [17] allowed us to characterize the alteration of synaptic plasticity in the pathological context cast by CSTB low level. As expected, low levels of CSTB had a strong impact on synaptic physiology. Indeed, in synaptosomes from EPM1 hCOs, the abundance of synaptic proteins was significantly diminished, with protein-specific profiles. In particular, patient synaptophysin remains lower than control at all stages, indicating a general impairment of the synaptic areas, while syntaxin and synaptotagmin 1/2 return to the control levels at 11 months, suggesting a delay in synaptic functions. Neurons generated in vitro from the EPM1 patients’ cells also displayed lower expression of synaptophysin compared to control. These data are in line with previous results indicating lower levels of synapsin 1 puncta in developing cerebellum from CSTB knockout mice, which is rescued at later stages [16].

To investigate the mechanism underlying the alteration of synaptic composition in EPM1 hCOs, we focused our attention to the synaptic system of protein synthesis [13]. In this regard, it has been recently emphasized the utilities of synaptosomes as a tool for studying the alterations of local protein translation in neuropathologies [46]. In particular, we analyzed eIF4G2, a component of eIF4G family [47] which is known to be synthesized in the axon and therefore bears crucial importance in modulating axonal protein synthesis and axonal growth [39]. We observed a relevant decrease of eIF4G2 in EPM1 synaptosomes, suggesting the impairment of synaptic system of protein synthesis in the pathology. On the other hand, eIF4G2 protein is more abundant in the EPM1 total lysate compared to control, suggesting that one of the pathological features of the disease is the alteration of transport mechanisms of different cargos, including translation-related proteins, from the soma to synaptic areas of the neurons. This hypothesis is supported also by the altered intracellular transport and protein synthesis machinery described in cerebellar synaptosomes from Cstb-KO mice [15]. It is noteworthy that CSTB is one of the proteins synaptically synthesized [14]. Therefore, the impairment of the synaptic system of protein synthesis in EPM1 may aggravate the CSTB deficit specifically in the synaptic area.

In line with the detected alteration in EPM1 synapses, we also observed a decrease of the EVs marker CD81 in the synaptosomes from patient hCOs. Mass spectrometry results corroborated these findings, showing depletion of proteins involved in secretion and vesicles transport in synaptosomes from EPM1 hCOs. These results suggest two possible scenarios related to the pathological low level of CSTB in EPM1: (1) lack of CSTB alters the secretion mechanism and trafficking of EVs in synaptic areas and (2) lack of secreted CSTB affects extracellular neurogenic environment leading to the pathology.

In addition to alteration in synaptic areas, EPM1 neurons exhibited early differentiation, in agreement with our previous results [17]. At later stages (10 weeks in culture), EPM1-derived neurons display also an altered morphology with longer and more branched, but thinner neurites. The altered neuronal complexity and reduced synapse density were previously shown also in iPSC-derived neurons from ASD patients [48].

In conclusion, our results suggest that EPM1 is characterized by early neuronal differentiation but delayed synaptic functions, which leads to synaptopathy and altered neuronal morphology and circuitry, manifesting an epileptic phenotype. Altogether, our data showing alteration of synaptic plasticity in EPM1 may open new venues toward the understanding of molecular mechanisms involving CSTB, which are impaired in the disease.

## Data Availability

The mass spectrometry proteomics data have been deposited to the ProteomeXchange Consortium via the PRIDE (Perez-Riverol et al., 2022, https://doi.org/10.1093/nar/gkab1038) partner repository with the dataset identifier PXD041125.
